# Variants in toll-like receptor 9 gene influence susceptibility to tuberculosis in a Mexican population

**DOI:** 10.1186/1479-5876-11-220

**Published:** 2013-09-21

**Authors:** Diana Torres-García, Alfredo Cruz-Lagunas, Ma Cecilia García-Sancho Figueroa, Rosario Fernández-Plata, Renata Baez-Saldaña, Criselda Mendoza-Milla, Rodrigo Barquera, Aida Carrera-Eusebio, Salomón Ramírez-Bravo, Lizeth Campos, Javier Angeles, Gilberto Vargas-Alarcón, Julio Granados, Radha Gopal, Shabaana A Khader, Edmond J Yunis, Joaquin Zuñiga

**Affiliations:** 1 Department of Immunolgy, Instituto Nacional de Enfermedades Respiratorias Ismael Cosío Villegas, Tlalpan 4502, Mexico City, 14080, Mexico; 2 Department of Epidemiology and Social Sciences, Instituto Nacional de Enfermedades Respiratorias Ismael Cosío Villegas, Mexico City, Mexico; 3 Department of Tuberculosis, Instituto Nacional de Enfermedades Respiratorias Ismael Cosío Villegas, Mexico City, Mexico; 4 Department of Pulmonary Fibrosis, Instituto Nacional de Enfermedades Respiratorias Ismael Cosío Villegas, Mexico City, Mexico; 5 Molecular Genetics Laboratory, National School of Anthropology and History, Mexico City, Mexico; 6 Jurisdicción Sanitaria No. 3 de Tuxtepec, Servicios de Salud del Estado de Oaxaca, Oaxaca, Mexico; 7 Department of Genomics, Instituto Nacional de Cardiología Ignacio Chávez, Mexico City, Mexico; 8 Department of Transplants, Instituto Nacional de Ciencias Médicas y Nutrición Salvador Zubirán, Mexico City, Mexico; 9 Division of Infectious Diseases, Children’s Hospital of Pittsburgh of UPMC, Pittsburgh, PA, USA; 10 Department of Cancer Immunology and AIDS, Dana Farber Cancer Institute, Harvard Medical School, Boston, MA, USA

**Keywords:** Tuberculosis, rs352139, TLRs, Gene polymorphisms, Susceptibility, TLR9

## Abstract

**Background:**

The control of *Mycobacterium tuberculosis* (Mtb) infection begins with the recognition of mycobacterial structural components by toll like receptors (TLRs) and other pattern recognition receptors. Our objective was to determine the influence of TLRs polymorphisms in the susceptibility to develop tuberculosis (TB) in Amerindian individuals from a rural area of Oaxaca, Mexico with high TB incidence.

**Methods:**

We carried out a case–control association community based study, genotyping 12 polymorphisms of TLR2, TLR4, TLR6 and TLR9 genes in 90 patients with confirmed pulmonary TB and 90 unrelated exposed but asymptomatic household contacts.

**Results:**

We found a significant increase in the frequency of the allele A of the TLR9 gene polymorphism rs352139 (A>G) in the group of TB patients (g.f. = 0.522) when compared with controls (g.f. = 0.383), (Pcorr = 0.01, OR = 1.75). Under the recessive model (A/G + A/A vs G/G) this polymorphism was also significantly associated with TB (Pcorr = 0.01, OR= 2.37). The association of the SNP rs352139 was statistically significant after adjustment by age, gender and comorbidities by regression logistic analysis (Dominant model: *p* value = 0.016, OR = 2.31; Additive model: *p* value = 0.023, OR = 1.68). The haplotype GAA of TLR9 SNPs was also associated with TB susceptibility (Pcorr = 0.02). Differences in the genotype or allele frequencies of TLR2, TLR4 and TLR6 polymorphisms between TB patients and healthy contacts were not detected.

**Conclusions:**

Our study suggests that the allele A of the intronic polymorphism rs352139 on TLR9 gene might contribute to the risk of developing TB in Mexican Amerindians.

## Background

Tuberculosis (TB) is a leading cause of death worldwide [1]. World Health Organization (WHO) estimates one-third of the world’s population is infected with *Mycobacterium tuberculosis* (Mtb) [2] and between the 2000 and 2020, about one billion people will become infected and 200 million people will develop active TB. Only 5 to 10% of the infected individuals develop the clinically active disease in their lifetime and the other 90% remain as latently Mtb infected individuals [2-4]. The progression to active TB is the result of the interplay between environmental, host genetic factors and pathogenic characteristics of the Mtb strain [3-6].

Multiple genes have been involved in the control of Mtb and progression to TB [7,8]. In this context, toll like receptors (TLRs) are a family of phylogenetically conserved genes, which are essential for recognition of a broad repertoire of pathogen-associated molecular patterns (PAMPs) on macrophages and dendritic cells and play an important role in the innate responses against Mtb [9-13].

Genetic variations of TLR1, TLR2, TLR4, TLR6 and TLR9 have been associated with the susceptibility to TB in different ethnic groups [14-20]. In contrast, other studies have failed to demonstrate significant associations of TLRs polymorphisms with TB [21-24].

To our knowledge, no previous studies have addressed the prevalence of TLRs polymorphisms in Mexican patients with TB. Therefore, we examined whether polymorphisms in TLR2, TLR4, TLR6 and TLR9 are associated with the susceptibility to pulmonary TB in Mexican individuals from a rural area with high incidence of TB.

## Materials and methods

### Subjects

Samples from 180 unrelated individuals (90 patients with diagnosis of pulmonary TB and 90 household healthy controls) were obtained. The diagnosis of pulmonary TB was based on the WHO criteria with presence of clinical symptoms, detection of acid-fast bacilli in sputum smear samples, Mtb positive cultures in Löwenstein-Jensen medium and X-ray evidence of cavitary lesions in lung. Only patients older than 18 years were included in the study. As a control group, we included unrelated individuals that were in close contact with TB patients, all of them were asymptomatic and no evidence of positive Mtb cultures or radiological lesions in lung were detected. Both, TB patients and controls were recruited from the programs of TB detection and control in the State of Oaxaca and were from the Mazatecan ethnic group and were living in the Town called Temascal, a rural area near from the City of Tuxtepec in Oaxaca State (a high pulmonary TB incidence area located in the southeast of Mexico). All studied individuals; their parents and grandparents were born in the Mazatecan area in Oaxaca State, Mexico. The clinical and demographic characteristics of TB patients and controls are shown in Table 1.

**Table 1:** Demographic and clinical characteristics of patients and controls

	**TB Patients**	**Controls**	
	**(N = 90)**	**(N = 90)**	P value*
**Age**	46.9 ± 17.8	42.9 ± 15.9	ns
**Gender**			
**M (%)**	38 (42.2)	32 (35.5)	ns
**F (%)**	52 (57.7)	58 (64.4)	ns
***Comorbidities***			
**Type 2 Diabetes n (%)**	16/90 (17.7)	0/90 (0)	<0.05
**Systemic hypertension n (%)**	4/90 (4.5)	2/90 (2.2)	ns
**Immunodeficiency n (%)**	1/90 (1.4)	0/90 (0)	ns
**Autoimmunity n (%)**	1/90 (1.4)	0/90 (0)	ns

Data are expressed as means ± SD, means and percentage or n and percentage.

*Comparisons of the continuous variables among groups were performed using the Mann–Whitney-Wilcoxon test, whereas the *X*^2^ test was used to compare frequencies.

The Institutional Review Board (IRB) of the National Institute of Respiratory Diseases (INER) reviewed and approved the protocol under which all subjects were recruited. All subjects provided written informed consent for these studies, and they authorized the storage of their DNA samples at INER repositories for this study.

### DNA isolation and TLR 2, 4, 6 and 9 SNP genotyping

Genomic DNA was isolated from EDTA-anticoagulated peripheral blood by using Qiagen blood mini kit (*Qiagen, Chatsworth, CA*). A total of 12 SNPs at the TLR2 (rs3804099, rs7656411, rs121917864, and rs5743708), TLR4 (rs5030710, rs5030725, rs12344353 and rs4986790), TLR6 (rs6815827) and TLR9 (rs352139, rs5743842, and rs5743836) genes were analyzed (See details in Table 2). The selection of these SNPs was based on their possible functional effect and due to existence of previous associations with infectious diseases. The SNPs genotyping was performed using the TaqMan 5’ nuclease assay. The PCR reaction was carried out using 15 ng of DNA, 12.5 ul of 2× TaqMan Universal PCR Master Mix, 0.625 ul of 40× Assay Mix and 8.8 ul of ddH_2_0. The genotyping was performed with validated TaqMan assays (*Life Technologies/Applied Biosystems, Foster City, CA*) TLR2 (C__22274563_10, C__29420880_10, Exp:06/2017 30 F04 and C__27860663_10), TLR4 (C__25761144_10, C___2704050_10, C__31784003_10 and C__11722238_20), TLR 6 (C__30687096_10) and TLR9 (C___2301953_10, C__30810101_10 and C__32645383_10). PCR conditions were: Hold 95°C/10 min, followed by 40 cycles of 92°C/15 s and 60°C/1 min. All PCR’s were performed using 96-well plates in a Step One plus real time PCR system (*Life Technologies/Applied Biosystems, Foster City, CA*).

**Table 2:** Characteristics of the studied SNPs at TLR2, TLR4, TLR6 and TLR9 genes

**Gene**	**Chr**	**SNP ID**	**Location**	**Genotype (Phenotype)**
TLR2	4	rs3804099	Exon (synonymous)	C> T (Asn199Asn)
		rs7656411	3′ near gene	G> T
		rs121917864	Exon (non-synonymous)	C> T (Arg677Trp)
		rs5743708	Exon (non-synonymous)	A> G (Arg753Gln)
TLR4	9	rs5030710	Exon (synonymous)	C> T (Ser105Ser)
		rs5030725	Intron	G> T
		rs12344353	Intron	C>T
		rs4986790	Exon (non-synonymous)	A/G (Asp299Gly)
TLR6	4	rs6815827	3′ near gene	C> T
TLR9	3	rs352139	Intron	A> G
		rs5743842	Exon	C> T (Arg5Cys)
		rs5743836	5′ near gene	C> T

### Statistical analysis

Demographic and clinical variables between TB patients and controls were analyzed with the Stata statistical software v8.0. We used a significance level of *p* <0.05. Power calculation showed that this significance level would yield a power of 80% with a sample size of 90 individuals per group. Hardy-Weinberg equilibrium was tested for all genotypic combinations of each SNP in TB patients and controls. The differences in the distribution of the allelic, genotypic and haplotype frequencies of TLR2, TLR4, TLR6 and TLR9 polymorphisms in TB patients and controls were evaluated by the Mantel-Haenszel, Chi-square test with 2x2 contingency tables using the EPIINFO statistical software v6.04b. *p* values resulting from the association analysis were corrected by the Bonferroni method. Relative risks (RR) with 95% confidence interval (CI) were estimated as the odds ratios (OR). The significance of the SNPs associated to TB was adjusted by age, gender and type 2 diabetes (T2D) by logistic regression analysis and the associations were tested under dominant and additive models. Pairwise LD was calculated by the genotype correlation coefficient (r^2^). For all pairs of autosomal SNPs, r^2^ measures were calculated using the software Haploview v4.2 [25].

## Results

Demographic and clinical characteristics of TB patients and controls are summarized in Table 1. Prevalence of T2D was significantly higher in TB patients (17.7%) when compared to controls (0%, p < 0.05). Mean age, gender and the prevalence of other comorbidities such as systemic hypertension, immunodeficiency and autoimmunity was similar among groups.

The gene and genotype frequencies of the TLR2, TLR4, TLR6 and TLR9 polymorphisms are presented in Table 3. Significant deviations from the Hardy-Weinberg equilibrium in the distribution of the TLRs SNP genotypes in TB patients and controls were not detected with the exception of the SNP rs121917864 of TLR2 (*X*^2^ = 88, p < 0.05 in both TB patients and controls).

**Table 3:** Distribution of TLR2, TLR4, TLR6 and TLR9 gene polymorphisms in patients with TB and controls

			**TB patients**		**Controls**		
			**(N=90)**		**(N=90)**		
	**SNP**	**Genotype**	**n**	**F**	**n**	**F**	**Pcorr**
		**Allele**					
**TLR2**	**rs3804099**	T/T	59	(0.656)	48	(0.533)	ns
		C/T	26	(0.289)	36	(0.400)	ns
		C/C	5	(0.056)	6	(0.067)	ns
		T	144	(0.800)	132	(0.733)	ns
		C	36	(0.200)	48	(0.267)	ns
	**rs7656411**	T/T	58	(0.644)	53	(0.589)	ns
		G/T	29	(0.322)	33	(0.367)	ns
		G/G	3	(0.033)	4	(0.044)	ns
		T	145	(0.806)	139	(0.772)	ns
		G	35	(0.194)	41	(0.228)	ns
	**rs121917864**	C/C	-	-	-	-	
		C/T	90	(1.0)	90	(1.0)	ns
		T/T	-	-	-	-	
		C	90	(0.500)	90	(0.500)	ns
		T	90	(0.500)	90	(0.500)	ns
	**rs5743708**	G/G	90	(1.0)	90	(1.0)	ns
		G/A	-	-	-	-	
		A/A	-	-	-	-	
		G	180	(1.0)	180	(1.0)	ns
		A	-	-	-	-	
**TLR4**	**rs5030710**	T/T	87	(0.967)	87	(0.967)	ns
		C/T	3	(0.033)	3	(0.033)	ns
		C/C	-	-	-	-	
		T	177	(0.983)	177	(0.983)	ns
		C	3	(0.017)	3	(0.017)	ns
	**rs5030725**	T/T	86	(0.978)	89	(0.989)	ns
		T/G	2	(0.022)	1	(0.011)	ns
		G/G	-	-	-	-	
		T	178	(0.989)	179	(0.994)	ns
		G	2	(0.011)	1	(0.006)	ns
	**rs12344353**	T/T	89	(0.989)	90	(1.0)	ns
		C/T	1	(0.011)	-	-	ns
		C/C	-	-	-	-	
		T	179	(0.994)	180	(1.0)	ns
		C	1	(0.006)	-	-	ns
	**rs4986790**	A/A	88	(0.978)	89	(0.989)	ns
		A/G	2	(0.022)	1	(0.011)	ns
		G/G	-	-	-	-	
		A	178	(0.989)	179	(0.994)	ns
		G	2	(0.011)	1	(0.006)	ns
**TLR6**	**rs6815827**	C/C	88	(0.978)	88	(0.989)	ns
		C/T	2	(0.022)	1	(0.011)	ns
		T/T	-	-	-	-	
		C	178	(0.989)	177	(0.994)	ns
		T	2	(0.011)	1	(0.006)	ns
**TLR9**	**rs352139**	**G/G**	**19**	**(0.211)**	**35**	**(0.389)**	0.01^*^
		A/G	48	(0.533)	41	(0.456)	ns
		A/A	23	(0.256)	14	(0.156)	ns
		**G**	**86**	**(0.478)**	**111**	**(0.617)**	0.01^**^
		**A**	**94**	**(0.522)**	**69**	**(0.383)**	0.01^***^
	**rs5743842**	T/T	-	-	-	-	
		T/C	2	(0.022)	-	-	ns
		C/C	88	(0.978)	90	(1.0)	ns
		T	2	(0.011)	-	-	ns
		C	178	(0.989)	180	(1.0)	ns
	**rs5743836**	T/T	82	(0.911)	78	(0.867)	ns
		T/C	8	(0.089)	12	(0.133)	ns
		C/C	-	-	-	-	
		T	172	(0.956)	168	(0.933)	ns
		C	8	(0.044)	12	(0.067)	ns

* OR = 0.42, 95% CI = 0.21-0.81.

** OR = 0.56, 95% CI = 0.38-0.86.

*** OR = 1.75, 95% CI = 1.15-2.67.

**ns:** non significant; **F:** frequency; **Pcorr:***p* values were corrected by Bonferroni. Statistically significant values are shown in bold.

We found a significant increase in the frequency of the allele A of the TLR9 gene polymorphism rs352139 (A>G) in the group of TB patients (gene frequency (g.f.) = 0.522) when compared to controls (g.f. = 0.383, Pcorr = 0.01, OR = 1.75, 95% CI = 1.15-2.67). Under a dominant model (A/A + A/G vs G/G), this polymorphism was associated with TB (Pcorr = 0.009, OR = 2.37, 95% CI = 1.22-4.60).

The frequency of the homozygous genotype G/G of the SNP rs352139 was considerably lower in the group of TB patients (g.f. = 0.211) compared to controls (g.f = 0.389) (Pcorr = 0.01, OR = 0.42, 95% CI = 0.21-0.81). Consequently the frequency of the allele G was significantly higher in the control group (g.f. = 0.617) than in TB patients (g.f. = 0.478) (Pcorr = 0.01, OR = 0.56, 95% CI = 0.38-0.86). We also analyzed the distribution of allele and genotype frequencies of the rs352139 polymorphism in males and females from the TB and control groups. A significant association of the allele A under a dominant model (A/A + A/G vs G/G) was found with TB in females (Pcorr = 0.00008, OR = 5.2 (95% CI = 2.3-12.0).

Importantly, the association of the genotypes of the SNP rs352139 with TB was confirmed by logistic regression analysis under the dominant (A/A + G/A vs G/G; p = 0.01, OR= 2.31, 95% CI= 1.50-4.63) and additive (2A/A + G/A, to G/G, p = 0.02, OR = 1.68, 95% CI = 1.07-2.65) models after adjustment for age, gender and T2D, Table 4.

**Table 4:** Association of the TLR9 polymorphism rs352139 (A>G) genotypes with pulmonary TB after logistic regression analysis

	**Genotype frequency (%)**						
	**G/G**	**G/A**	**A/A**	**MAF (A)**	**Model**	**OR (95% CI)**	**P value**
Control (n = 90)	0.389	0.456	0.156	0.383			
TB (n = 90)	0.211	0.533	0.256	0.522	Dominant (A/A + G/A vs G/G)	**2.31 (1.50-4.63)**	**0.016**
					Additive (2A/A + G/A, to G/G)	**1.68 (1.07-2.65)**	**0.023**


Associations were tested using logistic regression adjusting for Age, Gender and T2D.

**TB:** Tuberculosis; **MAF:** Minor allele frequency. Statistically significant values are shown in bold.

We also compared the distribution of all TLRs SNPs in TB patients without T2D and controls. The allele A of the SNP rs352139 was associated to TB in patients without T2D (Pcorr = 0.02, OR = 1.63, 95% CI = 1.06-2.57). Also, the dominant model (A/A + A/G vs G/G) was associated with TB, excluding individuals with T2D (Pcorr = 0.02, OR = 2.26, 95% CI = 1.12-4.55). Significant differences in the distribution of genotypes or alleles of the TLR9 polymorphisms (rs5743842 and rs5743836) were not detected.

The allele and genotype frequencies of the TLR2 (rs3804099, rs7656411, rs121917864, rs5743708); TLR4 (rs5030710, rs5030725, rs12344353, rs4986790) and TLR6 (rs6815827) polymorphisms were similar amongst TB patients and controls.

**Figure 1:**
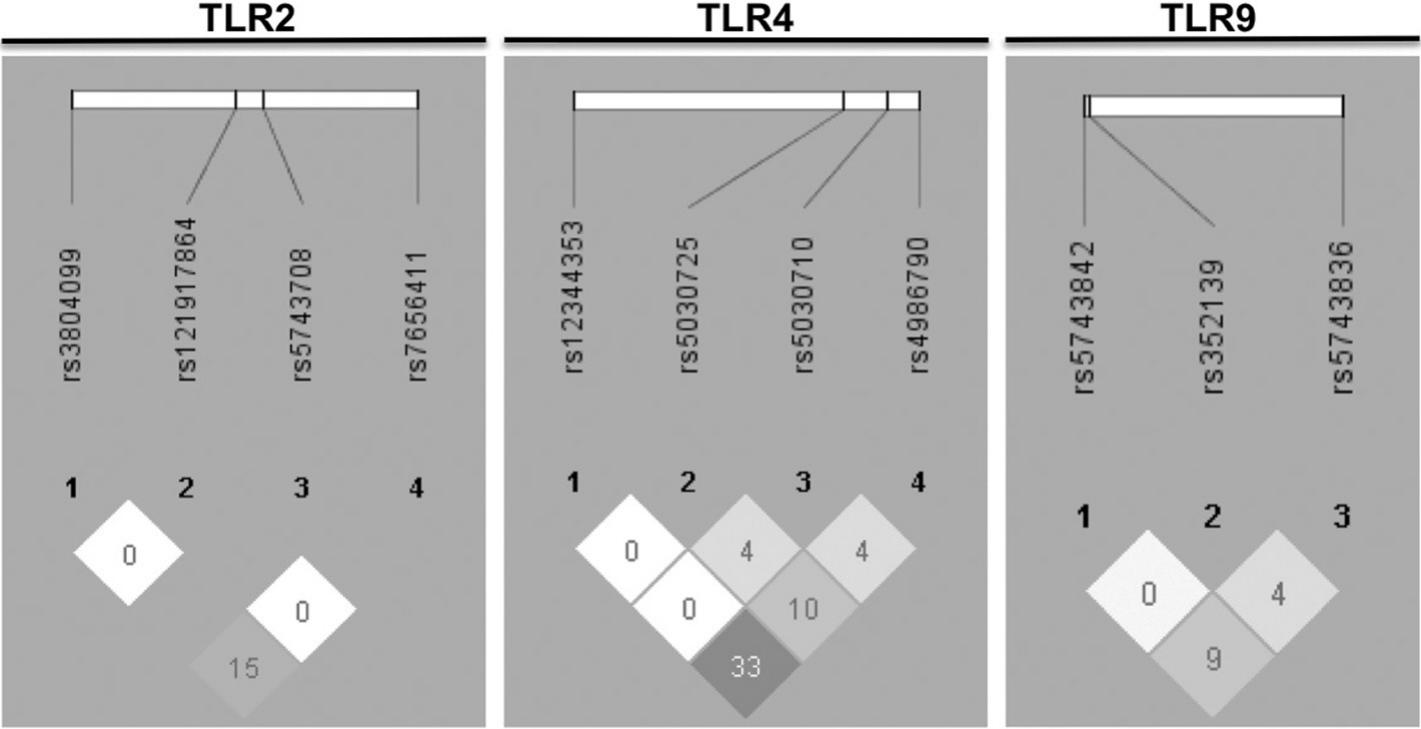
**Linkage disequilibrium (LD) plots of TLR2, TLR4 and TLR9 polymorphisms containing the significant single-nucleotide polymorphism of TLR9 (rs352139 (A>G) associated with pulmonary TB.** The LD plots were generated using Haploview v4.2 (Broad Institute, Cambridge, MA, USA) [25]. Numbers in squares indicate the correlation (r^2^) between SNPs.

The distribution of TLR2, TLR4 and TLR9 haplotypes in TB patients and controls are listed in Table 5. We found a significant association of the haplotype GAA of TLR9 with TB (TB patients: hf = 0.522 versus Controls: hf = 0.383; Pcorr = 0.02). No significant differences were detected in the distribution of TLR2 and TLR4 haplotypes between TB patients and controls. In general, low LD (r^2^) values were observed for all TLR2, TLR4 and TLR9 polymorphisms, Figure 1.

**Table 5:** Haplotype distribution of TLR2, TLR4 and TLR9 polymorphisms in patients with TB and controls

Haplotypes*	**TB patients**	**Controls**	***X***^**2**^	**Pcorr**
	**(N = 90)**	**(N = 90)**		
	**hf**	**hf**		
**TLR2**				
TTGT	0.349	0.324	0.243	ns
TCGT	0.349	0.324	0.243	ns
CTGG	0.046	0.071	1.053	ns
CCGG	0.046	0.071	1.053	ns
CTGT	0.054	0.062	0.103	ns
CCGT	0.054	0.062	0.103	ns
TTGG	0.051	0.042	0.154	ns
TCGG	0.051	0.042	0.154	ns
**TLR4**				
TTTA	0.972	0.972	0.0	ns
TTCA	0.011	0.017	0.203	ns
**TLR9**				
GGA	0.433	0.550	4.901	ns
** GAA**	**0.522**	**0.383**	**7.007**	**0.02**
GGG	0.033	0.067	2.105	ns

*Haplotypes: TLR2 [rs3804099, rs121917864, rs5743708, rs7656411].

TLR4 [rs12344353, rs5030725, rs5030710, rs4986790].

TLR9 [rs5743842, rs352139, rs5743836].

**ns:** non significant, **hf:** haplotype frequency. Statistically significant values are shown in bold.

## Discussion

Several studies have demonstrated that genetic factors play a major role in the susceptibility to TB [7,8]. Evidence suggests that polymorphisms within TLRs may cause an impairment of the immune response against TB; possibly modifying the TLRs affinity to Mtb derived ligands leading to alterations in the signal transduction of anti-Mtb immune responses [13,16]. These studies have been performed in different populations including Asians, African-Americans and Caucasians, but the results of genetic association studies are controversial [14-24]. Recent studies suggest that TLRs polymorphisms might not contribute markedly to TB susceptibility [21,26]. However, the current study provides evidence that TLRs polymorphisms contribute to pulmonary TB susceptibility in Mexicans. We found a significant association of the allele A of the SNP rs352139 (A>G) located in the intronic region of TLR9, on chromosome 3, with the susceptibility to TB in patients from a rural area in the State of Oaxaca in the southeast of Mexico. The association of this SNP was confirmed by logistic regression analysis, adjusting for age, gender and T2D. A significant association with the dominant A/A + A/G vs GG with TB was also detected and particularly with female. A decreased frequency of the homozygous genotype G/G of the SNP rs352139 was detected in the group of TB patients, suggesting a protective role of the allele G. Moreover, the TLR9 haplotype containing the allele A of this SNP (Haplotype GAA) was associated with the susceptibility to TB.

TLR9 is expressed by different cells of the immune system and is essential in the activation of the innate responses against Mtb infection [9]. TLR9 recognizes unmethylated CpG DNA motifs and functional studies revealed that the binding of TLR9 is necessary to drive the Th1 immune response [27]. The importance of TLR9 in the antimycobacterial responses has been supported in experimental models using the TLR9-deficient mice [9,10].

In line with our results, the allele A of the SNP rs352139 has been strongly associated with susceptibility to TB in African-Americans but not with Caucasians and African patients from Guinea-Bissau [17]. Functional studies suggest that the intronic TLR9 polymorphism rs352139 A>G exerts a regulatory effect of TLR9 gene expression. The presence of G allele and the C allele of the promoter polymorphism −1486, is associated with downregulation the TLR9 expression at the transcriptional level. In contrast, the presence of the allele A (in presence of the allele T of the promoter polymorphism T allele at −1486) induces the up-regulation of TLR9 expression [28]. It is possible that this variation influences signaling by creating an alternative splicing site, affecting the TLR9 mRNA transcription. Otherwise, the rs352139 SNP could be a likely marker in LD with a polymorphic regulatory region that controls TLR9 expression or a functional coding region polymorphism [28]. However, the effect of this intronic SNP in the induction or downregulation of TLR9 expression is not well defined, particularly in the context of infectious diseases.

Different from other pathogens, Mtb infection can persist in the host for long periods in a dormant or latent state, even in a fully functioning immune system [29]. TB immunity is mediated by Th1-type responses; nevertheless this response is apparently not enough to definitively eradicate the chronic infection or the latent bacilli [30]. It is possible that the presence of the allele A of the rs352139 SNP in individuals with latent TB infection may result in a high TLR9 expression that contribute to the induction of high levels of proinflammatory cytokines. A pro-inflammatory environment in lung confers protection against Mtb infection. However, a pathogenic or exacerbated pulmonary inflammation may exert deleterious effects [31], promoting the infiltration inflammatory cells to the lung that become Mtb infected. A dysregulated inflammatory environment, potentially can also contribute to the tissue damage and dissemination of the mycobacteria to other areas of the lung and peripheral tissues. It is important to remark that the allele A of this polymorphism of TLR9 has been associated with the susceptibility and severity of parasitic and viral infections [32,33].

Interestingly, the polymorphism rs352139, located in the intronic region of *TLR9*, has been associated with the susceptibility to TB in Indonesian females [14]. Besides, it is noteworthy that this polymorphism was associated with pulmonary TB in females from South India [34]. In our study, we found a significant association of this polymorphism, particularly the homozygous A/A and heterozygous A/G genotypes, with the susceptibility to TB in females. The reason why these genotypes contribute to the susceptibility to TB in females is not fully clear. In this regard, previous studies have suggested that significant differences exist in TB incidence between males and females [35,36]. The physiological hypothesis of infectious diseases susceptibility, suggest that differences in the expression of sex hormones and genetic variability between males and females might contribute in the differential incidence of infectious diseases among them [37]. Moreover, *in vitro* studies have revealed that progesterone regulates the production of type I interferon (IFN-α), possibly through the interference of MyD88-dependent activation of interferon regulatory factor 7 (IRF-7) after stimulation with the TLR9 ligand CpG in both, mice and humans. Consequently, the inhibitory effect of this female sex steroid hormone on IFN-α production may impair inflammatory responses and pattern recognition receptor signaling against pathogens [38].

From the population genetics perspective, it is interesting that this SNP is associated with susceptibility to TB across Asian and Amerindian groups. The Mazatecans inhabit the mountains of northern Oaxaca (Mexico). They belong to the ancient Central-American cultural groups and may be related to the Olmecs (the most ancient Meso-American culture). The first Amerindian Natives are believed to have come from Asia through the Bering land bridge between 30,000–12,000 years before the present. These assumptions have been based on cultural, morphological and genetic connections between Native American and Asian populations [39-41]. Supporting the hypothesis of the presence of Asian genes in Amerindian populations, in previous published studies we have detected Asian haplotypes (HLA-DRB1*15:02-DQB1*0602) in Mazatecans [42]. Also, a short genetic distance between Chinese and Native Americans was detected using Alu insertions data [43]. Anthropological genetic studies have suggested that some Asian genes may have been introduced to Native Americans by a Trans-Pacific route of migration from South East Asian populations [44,45].

Our study has some limitations, including its relatively small sample size, restricted by the study’s focus on patients from Amerindian ancestry with a recent diagnosis of pulmonary TB. In addition, we chose not to include individuals with familial history of autoimmune and neoplastic disorders. The second limitation is the lack of functional assays to determine the effect of TLR9 variation in the susceptibility to pulmonary TB. The strengths of our study includes the analysis of the influence of TLRs polymorphisms in the susceptibility to TB in a well genetically characterized ethnic group of Mexican Amerindians and the replication of the associations of the TLR9 intronic polymorphism previously described in other Asian ethnic groups.

## Conclusions

In summary, our findings suggest that the allele A of the polymorphism rs352139, located in the intronic region of the TLR9 gene could be implicated in the susceptibility to TB in Mazatecan Amerindians from the Oaxaca State in Mexico. Additional studies in a large number of TB patients and functional studies may help to establish the true significance and the possible deleterious effect of this polymorphism in the susceptibility to pulmonary TB.

## Abbreviations

g.f.: Gene frequency; INER: Instituto Nacional de Enfermedades Respiratorias Ismael Cosío Villegas; Mtb: *Mycobacterium tuberculosis*; PAMPs: Pathogen associated molecular patterns; RR: Relative risk; T2D: Type 2 diabetes; TB: Tuberculosis; TLRs: Toll like receptors; WHO: World health organization.

## Competing interests

All authors declare that they have no competing interests.

## Authors’ contributions

JZ, SAK, GVA EJY, CGS, ACL and CMM participated in the design of this study. JZ, JG, GVA, EJY, SAK and CGS participated in the design and coordination of the study and drafted the manuscript. DTG, ACL, LC and RG carried out the DNA isolation and genotyping. JZ, DTG, ACL, ACE, SRB and JG collected the peripheral blood samples. JZ, SAK, RG, JA, GVA, CMM, ACL, RBS, CGS and RFP performed the data analysis. JZ, CGS, RFP and JA performed the statistical analysis. This work was submitted in partial fulfillment of the requirements to obtain the PhD degree for DTG at Biomedical Sciences, Facultad de Medicina, Universidad Nacional Autónoma de México. All authors read and approved the final manuscript.
